# Effect of Recombinant Prophenin 2 on the Integrity and Viability of *Trichomonas vaginalis*

**DOI:** 10.1155/2015/430436

**Published:** 2015-03-01

**Authors:** J. L. Hernandez-Flores, M. C. Rodriguez, A. Gastelum Arellanez, A. Alvarez-Morales, E. E. Avila

**Affiliations:** ^1^Unidad Irapuato, Departamento de Ingeniería Genética, Centro de Investigación y de Estudios Avanzados del IPN, P.O. Box 629, 36500 Irapuato, GTO, Mexico; ^2^Division de Ciencias Naturales y Exactas, Departamento de Biologia, Universidad de Guanajuato, Col. Noria Alta, 36040 Guanajuato, GTO, Mexico; ^3^Universidad Politécnica del Mar y la Sierra, Carretera a La Cruz km 15.5, Col. Arroyitos, La Cruz, 82700 Elota, SIN, Mexico

## Abstract

*Trichomonas vaginalis* is the causal agent of trichomoniasis, which is associated with preterm child delivery, low birth weight, and an increased risk of infection by human papilloma virus and human immunodeficiency virus following exposure. Several reports have established increasing numbers of trichomoniasis cases resistant to metronidazole, the agent used for treatment, and it is therefore important to identify new therapeutic alternatives. Previously, our group reported the effect of tritrpticin, a synthetic peptide derived from porcine prophenin, on *T. vaginalis*; however, the hemolytic activity of this small peptide complicates its possible use as a therapeutic agent. In this study, we report that the propeptide and the processed peptide of prophenin 2 (cleaved with hydroxylamine) affected the integrity and growth of *T. vaginalis* and that pro-prophenin 2 displays some resistance to proteolysis by *T. vaginalis* proteinases at 1 h. Its effect on *T. vaginalis* as well as its low hemolytic activity and short-time stability to parasite proteinases makes prophenin 2 an interesting candidate for synergistic or alternative treatment against *T. vaginalis*.

## 1. Introduction

Antimicrobial peptides (AMPs) are natural antibiotics synthesized by all known organisms, from bacteria to vertebrates; they have antimicrobial and immunoregulatory functions. AMPs are active against several infectious agents, including viruses, bacteria, fungi, and parasites [1–3]. Most studies have examined the effects of AMPs on bacteria, and fewer reports exist regarding their effects on protozoa [3–8]. In mammals, defensins and cathelicidins are AMPs that are widely expressed in phagocytic immune cells that migrate to infection sites [9].

Pigs have the most diverse collection of cathelicidins of any mammalian species, among which are the proline-phenylalanine-rich prophenin-1 (PF-1), prophenin-2 (PF-2), proline-arginine-rich PR-39, and cysteine-rich protegrins 1 to 5 (PG-1 to PG-5), which have been purified from neutrophils [10].

The protozoan flagellate* Trichomonas vaginalis* is the causal agent of trichomoniasis, the most common nonviral sexually transmitted disease. Trichomoniasis is associated with membrane rupture in pregnant women and, therefore, preterm child delivery, low birth weight, and augmented risk of infection with human papilloma virus or human immunodeficiency virus type 1 [11–14] following exposure. Trichomoniasis is treated with metronidazole or tinidazole, but several reports describe increasing numbers of cases resistant to these compounds [15–19]. AMPs are potential alternatives for trichomoniasis treatment, but* T. vaginalis* trophozoites are rich in cysteine proteinases that might degrade the peptides; some of these enzymes are surface associated, excreted/secreted, and involved in parasite adherence and cytopathogenicity [20–22].

We reported an interesting trichomonacidal effect of tritrpticin, a small peptide derived from the porcine prophenin 2 [23], but the complete prophenin 2 peptide has not been tested for activity against* T. vaginalis* or other protozoan pathogens. In addition, prophenin 2 is interesting because it should theoretically be resistant to the attack by* T. vaginalis* proteinases due to its high proline content, which may prevent cleavage of the peptide bonds [10, 24]. In this study, we demonstrated that pro-prophenin 2 is partially resistant to proteolysis by* T. vaginalis* proteinases at 1 h and that the complete propeptide and the processed prophenin 2 peptide diminished the integrity and growth of* T. vaginalis*.

## 2. Materials and Methods

### 2.1. Strains and Plasmids

Plasmids were propagated and maintained using* E. coli* DH5*α* and purified using a Plasmid Midi Kit 100 (Qiagen, Cat. 12145). For protein expression,* E. coli* BL21 STAR was used. Both strains were grown at 37°C in Luria-Bertani medium supplemented with 100 mg/L carbenicillin (LB/Cb) when necessary. The plasmid pQE-TriSystem (Qiagen, Cat. 33903) was used to express pro-prophenin 2.

*T. vaginalis* strain RFC-1 (ATCC) was grown in 8 mL screw-cap tubes with 5 mL of TYI-S-33 medium, pH 7.0 [25], supplemented with 6% bovine serum.* T. vaginalis* was cultured for 72 h at 36.5°C to maintain trophozoites and for 24 h before assays.

### 2.2. Prophenin 2 Propeptide Cloning and Expression

**Figure 1:**
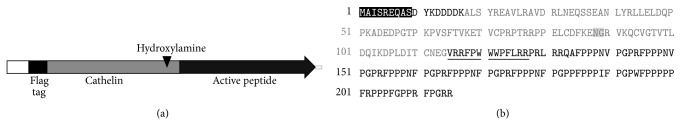
Amino acid sequence of pro-prophenin 2. (a) Graphical representation of the DNA fragment cloned into the pQE-TriSystem. (b) Amino acid sequence. Amino acids derived from the vector (white), Flag tag (black), cathelin (light gray), and processed peptide (dark gray). In (b) the hydroxylamine cleavage site (shaded) and the tritrpticin sequence (underlined) are shown.

The nucleotide sequence of the open reading frame corresponding to porcine pro-prophenin 2 [26] was optimized for* E. coli* codon usage. The optimized open reading frame was fused to an 8-amino acid FLAG tag [27] (Figure 1) followed by Western blot analysis and purification of the recombinant protein by affinity chromatography. The optimized pro-prophenin 2::FLAG tag was synthesized (Epoch Biolabs), cloned into the SmaI-EcoRI sites of the pQE-TriSystem expression vector and transformed into* E. coli* DH5*α*. Putative recombinant clones were confirmed by restriction analysis and sequencing. Confirmed clones were propagated using* E. coli* DH5*α*, and plasmid DNA was isolated; one clone, pUG2, was selected. To express pro-prophenin 2,* E. coli* BL21 STAR was transformed with the pUG2 construct and grown in LB/Cb. Overnight cultures were used to inoculate fresh LB/Cb medium supplemented with 0.4% glucose and incubated at 37°C until the OD600 reached 0.5. IPTG was then added to a final concentration of 0.25 mM, and the cells were further incubated for 4 h at 37°C. Cultures were centrifuged at 5,000 ×g for 20 minutes at 4°C. Cell pellets were washed twice with buffer A (100 mM Tris pH 7.5, 150 mM NaCl) and were stored at −80°C until used.

### 2.3. Pro-Prophenin 2 Purification

Bacterial pellets were thawed and kept on ice, then 1 mg/mL lysozyme and 25 *μ*g/mL DNase I were added and incubated 30 minutes at 37°C and disrupted with a VirTis sonicator (model VirSonic 60) using 6 pulses of 30 seconds each with 30-second intervals and a 5-watt output. The soluble phase was recovered by centrifugation at 14,500 ×g for 20 minutes and further clarified by filtering through a 0.2 *μ*m syringe filter (Nalgene, Cat. 190-9920). A total of 300 *μ*L of anti-Flag M2 affinity gel (Sigma, Cat. A2220) was applied to the supernatant and incubated overnight at 4°C with gentle agitation. Anti-Flag M2 affinity gel was recovered and washed 3 times with 2 mL of TBS (50 mM Tris-HCl, 150 mM NaCl pH 7.5)/0.02% NaN_3_, and pro-prophenin 2 was eluted with three 6 mL aliquots of 0.1 M glycine-HCl, pH 3.5, into Eppendorf tubes containing 20 *μ*L of 1 M Tris-HCl, pH 8.0. Anti-Flag M2 affinity gel was reequilibrated to neutral pH using 30 volumes of TBS. Pro-prophenin 2 was dialyzed using 14 kDa cutoff dialysis tubing (Spectra/Por, Cat. 132678) and concentrated using a Concentrator plus (Eppendorf, Cat. 5305 000.304).

### 2.4. Hydroxylamine Cleavage of Pro-Prophenin 2

To release the processed peptide, the purified pro-prophenin 2 was incubated 4 h at 45°C in a hydroxylamine reaction mixture (0.22 M Tris, 1.7 M hydroxylamine hydrochloride, 4 M guanidine hydrochloride, and pH 9). After incubation, the reaction mixture was incubated in an ice bath for 20 minutes and desalted using an Excellulose GF-5 Desalting Column (Pierce, Cat. 1851850); 10 fractions of 1 mL each were collected and concentrated using a Concentrator plus (Eppendorf, Cat. 5305 000.304). Because hydroxylamine cleavage is incomplete, uncleaved propeptide and cathelin were removed by affinity chromatography, using anti-Flag M2 affinity gel as described for pro-prophenin 2 purification.

### 2.5. Hemolytic Assay

To determine the hemolytic activity of the peptides, fresh human red blood cells were used; peripheral blood was collected from healthy volunteer donors into Vacutainer tubes containing heparin (Cat. 366480) or EDTA (Cat. 366450). Erythrocytes were washed three times with phosphate-buffered saline (PBS, 2.7 mM KCl, 10 mM Na_2_HPO_4_, 1.8 mM KH_2_PO_4_, 150 mM NaCl, and pH 7.0) and resuspended in PBS at the originally collected blood volume (erythrocyte concentration of 100%). The assay was performed in sterile 96-well microtiter plates. The final reaction volume was 200 *μ*L containing 1% erythrocytes and different concentrations of pro-prophenin 2, processed peptide, and amidated tritrpticin. Plates were incubated for 1 h at 37°C and centrifuged at 1000 ×g for 5 min. Released hemoglobin was determined by measuring absorbance at 540 nm in the supernatant. Erythrocyte integrity was calculated according to the hemoglobin released from a 100% hemolysis control (red cells lysed with 0.1% Triton X-100) and a negative control (red cells incubated with PBS), using the following formula: % hemolysis = [(*λ*_540_ in  peptide  solution − *λ*_540_ PBS)/(*λ*_540_  0.1%  Triton  X-100 − *λ*_540_  PBS)] × 100.

### 2.6. Peptide-Trichomonas Interactions

To determine the effect of the propeptide and processed prophenin 2 on the integrity and growth of* T. vaginalis*, peptide-trichomonas assays were performed, as previously described [23]. Briefly,* T. vaginalis* RFC-1 was grown for 24 h in screw-cap tubes containing 5 mL of  TYI-S-33. Cell were collected by centrifugation at 1000 ×g for 5 min and washed with 5 mL of TYI. The* T. vaginalis*-peptide interaction took place in 96-well sterile microtiter plates (Corning Inc., USA) containing 40,000 trophozoites and different concentrations of peptide in a final volume of 100 *μ*L. The medium for the 4 h interaction assay was TYI supplemented with 25 mM sodium bicarbonate and 100 mM HEPES, pH 7.0. Complete TYI-S-33 culture medium was used for the 24 h interaction assays. The cultures were incubated at 36.5°C in a microaerophilic atmosphere. After incubation, the trophozoite number was calculated using a Neubauer chamber and compared with a control group that did not interact with the peptides. The effect of the different peptide concentrations on* T. vaginalis* was evaluated by ANOVA and a mean comparison using the least significant difference (LSD) test (*α* = 0.05). Using the data obtained, we estimated the LD_50_ (lethal dosage 50) for the 24 h interaction, adjusting the model as follows: PERCENT INHIBITION/MORTALITY = A ∗ log (DOSAGE) + B, using the lm function in the R environment.

### 2.7. Stability of Pro-Prophenin 2 to* T. vaginalis* Proteinases

The stability of pro-prophenin 2 was determined at 1, 2, and 4 h of interaction with* T. vaginalis* in TYI supplemented with 25 mM sodium bicarbonate and 100 mM HEPES, pH 7.0, as described above. A 5 *μ*M final concentration of pro-prophenin 2 was added to 40,000* T. vaginalis* trophozoites in a volume of 100 *μ*L; after incubation at 37°C, samples were analyzed by Western blot using an anti-Flag antibody. A similar set of samples was incubated in the presence of the cysteine proteinase inhibitor E64.

## 3. Results

### 3.1. Pro-Prophenin 2 Expression and Purification

**Figure 2:**
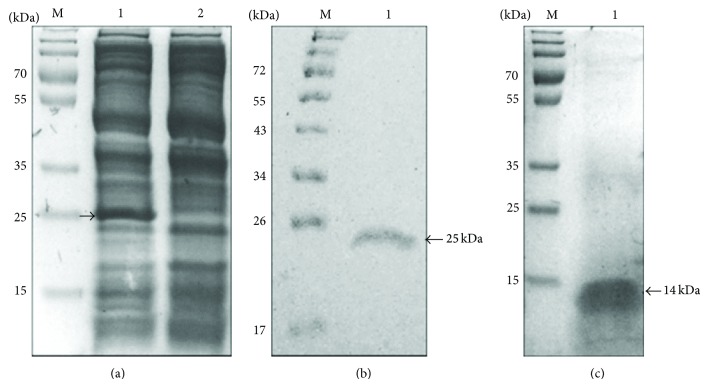
Electrophoretic analysis of pro-prophenin 2 expression, purification, and cleavage with hydroxylamine. Twelve percent polyacrylamide gels (20 : 1 acrylamide : bis-acrylamide) were used and proteins were stained with Coomassie blue. (a) Pro-prophenin 2 expression: lane 1, crude extract from* E. coli* BL21 STAR induced 4 h with 0.25 mM IPTG; lane 2, crude extract from uninduced* E. coli* BL21 STAR, otherwise grown as induced. (b) Pro-prophenin 2 purified by affinity chromatography (lane 1). (c) Processed prophenin 2 after hydroxylamine cleavage and purification (lane 1). M: molecular weight markers.

To express pro-prophenin 2, a synthetic gene was obtained and cloned into the pQE-TriSystem vector. We verified the correct in-phase insertion of the pro-prophenin 2 open reading frame into the expression vector by restriction analysis and sequencing (LANGEBIO, Irapuato, Mexico). Transformed* E. coli* induced with 1 mM IPTG expressed a protein with the expected molecular weight of the FLAG pro-prophenin 2 fusion protein; however, all of the recombinant protein was found in the pellet. Soluble pro-prophenin 2 was obtained by inducing the cultures with 0.25 mM IPTG, followed by 4 h incubation at 37°C after induction (Figure 2(a), lane 1).

FLAG pro-prophenin 2 was purified by affinity chromatography with an anti-FLAG M2 antibody using a wash buffer containing 300 mM NaCl and 3% Tween 20 and exhaustively washing the column to avoid copurification of contaminant proteins (Figure 2(b)). Pro-prophenin 2 yield was 325 *μ*g per liter of culture on average.

To obtain the processed portion of prophenin 2, the propeptide was cleaved with hydroxylamine; the cleavage site is located within the cathelin region, 26 amino acid residues upstream the predicted mature peptide (Figure 1(b), shaded residues). Pro-prophenin 2 cleavage with hydroxylamine had an efficiency of approximately 60% (data not shown). Using an anti-FLAG immunoaffinity column, processed prophenin 2 eluted in the void volume as a single band without binding to the antibody (Figure 2(c)), whereas uncleaved pro-prophenin 2 as well as the cathelin region was retained.

### 3.2. Hemolytic Assays

**Figure 3:**
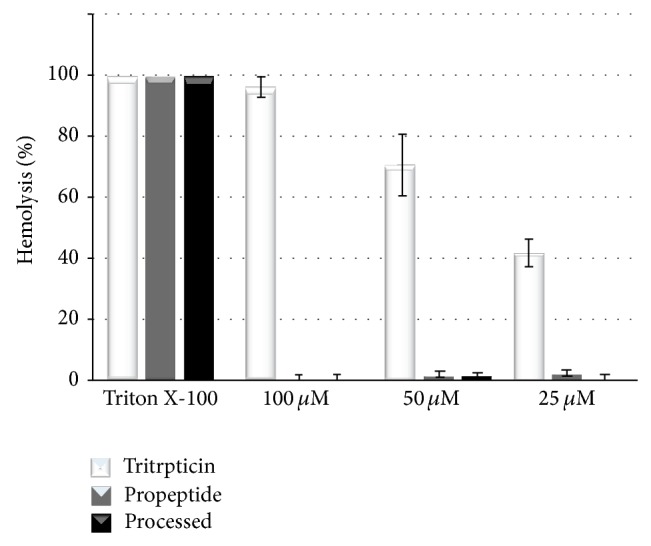
Hemolytic activity of prophenin 2. The percentage of hemolysis was calculated from the absorbance at 540 nm of the supernatants after 1 h of interaction of red blood cells with pro-prophenin 2, processed peptide, and NH_2_-tritrpticin. Hemolysis of 100% was obtained by the treatment of erythrocytes with 0.1% Triton X-100.

To determine the hemolytic effect of pro-prophenin 2 and the processed peptide, we performed interaction experiments using the peptides pro-prophenin 2, processed prophenin 2, and tritrpticin with human red blood cells.  Figure 3 shows the integrity of the erythrocytes after 1 h of interaction with different concentrations of peptides. We observed almost no effect of either the processed peptide or pro-prophenin 2 on human erythrocytes compared with erythrocytes treated with 0.1% Triton X-100 (100% hemolysis control). Hemolytic activity of NH_2_-tritrpticin is shown as control.

### 3.3. Peptide-Trichomonas Interactions

**Figure 4:**
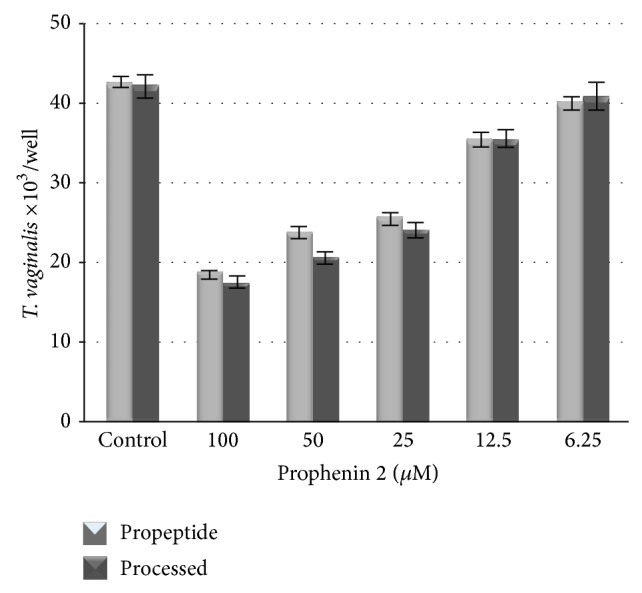
Effect of prophenin 2 on* T. vaginalis* integrity. Trophozoites were inoculated in TYI-HEPES-sodium bicarbonate at 40,000 cells/well; serial dilutions of prophenin 2 were added and incubated at 36.5°C for 4 h. After incubation, each well was sampled and trophozoites were counted. The size of the sample for every treatment was *n* = 44–48, and the standard errors observed were 1.18 or less.

The effect of prophenin 2 on* T. vaginalis* was estimated using a 4 h-interaction assay to examine trophozoite integrity, and a 24 h assay was used to assess parasite growth. After 4 h incubation with prophenin 2, refringent trophozoites were decreased by 3% at 6.25 *μ*M to 58% at 100 *μ*M of the processed peptide, showing a dose-dependent effect (Figure 4). Surprisingly, pro-prophenin 2 also had a deleterious effect on* T. vaginalis* integrity, and the effect was dose dependent, similar to the processed peptide (Figure 4).

**Figure 5:**
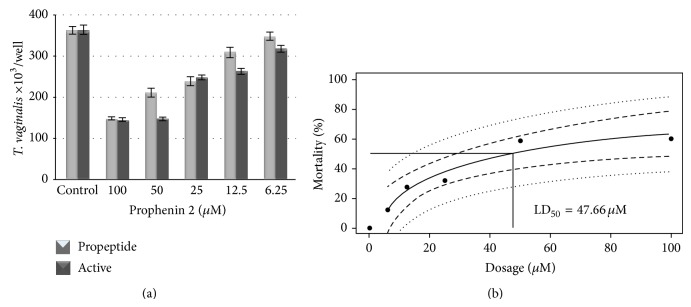
Effect of prophenin 2 on* T. vaginalis* growth. (a) Trophozoites were inoculated in TYI-S-33 at 40,000 cells/well; serial dilutions of pro-prophenin 2 were added and incubated at 36.5°C for 24 h. After incubation, each well was sampled, and trophozoites were counted. The size of the sample for every treatment was *n* = 44–48, and the standard errors observed were 1.18 or less. (b) Estimated LD_50_ for prophenin 2. The results from 24 h interaction assays were used to construct a linear regression model, and the LD_50_ was estimated to be 47.66 *μ*M.

*T. vaginalis* grew less at 24 h in the presence of pro-prophenin 2 or processed peptide compared with the culture in the absence of the peptides. Growth was reduced by 12% at 6.25 *μ*M and by 60% at 100 *μ*M of the processed peptide (Figure 5(a)). The results demonstrated high reproducibility because the standard error was low (11.24). Comparing the means of every treatment, we observed a dose-dependent effect on the integrity and growth of* T. vaginalis* and found that the LD_50_ is approximately 50 *μ*M of the processed peptide. Based on the linear regression analysis of the equation described in Section 2, we determined that the LD_50_ for the processed peptide was 47.66 *μ*M (Figure 5(b)). The significance level of the regression was *P* = 2.2*e* − 16, indicating an effect on the growth of* T. vaginalis*.

### 3.4. Stability of Pro-Prophenin 2 to* T. vaginalis* Proteinases

**Figure 6:**
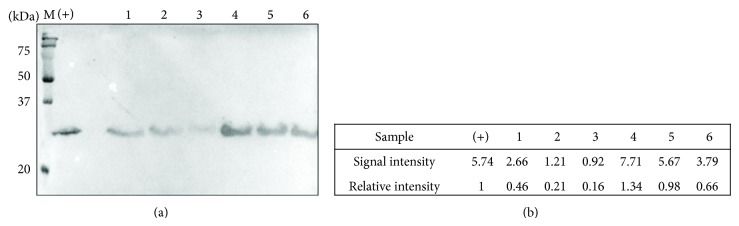
Stability of pro-prophenin 2 after interaction with* T. vaginalis*. (a) After parasite-peptide interaction at several incubation times, contents of wells (cells + supernatant) were analyzed by Western blot using anti-FLAG M2 antibodies and signal developed with luminol. Lane M: molecular weight markers; lane (+): 5 *μ*M pro-prophenin 2 as a positive control. Lanes 1 to 3:* T. vaginalis* with 5 *μ*M pro-prophenin 2 incubated 1, 2, and 4 h, respectively. Lanes 4 to 6: same as lanes 1 to 3 but in the presence of 10 *μ*M E64. (b) Signal intensity analysis is done with Image Lab software (BioRad).

Previous work in our group demonstrated that human cathelicidin LL-37 is very sensitive to proteases from* E. histolytica*, being degraded in just one hour [28]. We decided to test the stability of pro-prophenin 2 by Western blot of the propeptide after 1, 2, and 4 h of interaction with* T. vaginalis*, using the anti-FLAG M2 antibody. We detected the presence of pro-prophenin 2 in all lanes (Figure 6), even in samples that were not treated with the cysteine proteinase inhibitor E64; although after 4 h interaction, without E-64, the signal was very weak (lane 3).

## 4. Discussion

This study demonstrated that recombinant prophenin 2, both the propeptide and the processed peptide, diminished the integrity and growth of* T. vaginalis*. Prophenin 2 belongs to the porcine cathelicidin family, which are host defense peptides sharing a highly conserved N-terminal cathelin region homologous to the cathepsin L inhibitor [29]. Pigs possess the largest number of cathelicidins described for a single species, with 11 members [10, 30]. Originally, the sequence of prophenin was described by Pungercar et al. in 1993 from a cDNA clone [31]. Later, the protein prophenin-1 was purified and characterized as a 79 aa fragment from porcine leukocytes. It is rich in proline and phenylalanine and contains 5 nearly perfect tandem repeats of a proline-rich decamer, FPPPNFPGPR [32]. Additionally, Zhao et al. [26] reported a closely related sequence named prophenin 2. To date, much of the work performed with prophenin has focused on a small derivative named tritrpticin, which is 13 amino acid residues long and possesses 3 consecutive tryptophan residues. Most studies have used bacteria [33, 34], and only our group has reported the effect of tritrpticin on a protozoa [23]. In this work we expressed the recombinant pro-prophenin 2; although the yield was low (about 300 *μ*g/L), this can likely be circumvented using vectors that fuse pro-prophenin 2 to thioredoxin or glutathione S transferase; we are currently pursuing this line of research.

The use of vital staining to determine viability of* T. vaginalis* in studies involving interaction with antimicrobial peptides is not a suitable methodology, since these molecules alter the cell membrane making it more permeable to the colorant [4]. In a previous report, we demonstrate that after treatment with tritrpticin, the number of refringent* T. vaginalis* trophozoites correlates with their ability to growth [23]. Additionally we observed that tritrpticin at 105 *μ*M reduced* T. vaginalis* viability by approximately 72% after 3 h of interaction and that it reduced growth by 58% after 24 h of interaction [23]. In this study, we reported similar results, with a reduction of 58% in* T. vaginalis* integrity at 100 *μ*M of processed prophenin 2 in 4 h interaction assays and a decrease of 60% in* T. vaginalis* growth using 100 *μ*M of processed peptide in 24 h interaction assays.

A major concern regarding the possible therapeutic use of C-terminus amidated tritrpticin is the hemolytic activity, as it demonstrates ~50% hemolysis at 30 *μ*M [35]. Our work demonstrated that prophenin 2 presents very low hemolytic activity against human red blood cells. This hemolytic activity has to be determined on an individual case basis because it depends not only on the surface selectivity of the target membrane based on the lipid composition [36], but also on the composition of the peptide itself [37]. We demonstrated that prophenin 2 presented good trichomonacidal activity and very low hemolytic activity, which makes this molecule an attractive candidate for studying its possible use as a synergistic or alternative therapy.

In nature, it is accepted that prophenin 2 is processed by neutrophil elastase [38, 39]; the proteolytic cleavage site is predicted between Gly114 and Val115 to release a 101 aa mature peptide [26]. In this study we used as the processed prophenin 2, the portion obtained by cleavage of the recombinant propeptide at a naturally occurring hydroxylamine site (cleavage site Asp88-Gly89), which contained 26 extra amino acids at the N-terminus of the predicted mature peptide. We do not know if these extra amino acids affect the activity of the peptide because the antimicrobial activity of the mature peptide of prophenin 2 is unknown. However, the 79-80 and 17-18 aa prophenin peptides isolated from porcine leukocytes [32, 40, 41] have antibacterial activity. The predicted site for processing the propeptide includes 18 amino acids more than the 79 aa peptide isolated from leukocytes [26]; these fragments and the 17-18 aa long peptide may be produced by additional processing of the mature peptide.

In porcine leukocytes, prophenins are amidated at the C-terminus [40, 41], and this extra positive charge may contribute to the binding of negatively charged targets; therefore, it is expected that the amidated version of the peptide tested in this study will be active at lower concentrations against* T. vaginalis*.

Interestingly, our results demonstrate similar anti-*T. vaginalis* activity of recombinant pro-prophenin as the processed peptide, which is in accordance with the observation that the human pro-cathelicidin HCap18 has similar antimicrobial activity than the C-terminal LL-37 active peptide [42].

*T. vaginalis* secretes cysteine proteinases as virulence factors, and these enzymes may negatively affect the integrity of most antimicrobial peptides. It is proposed that the proline-rich sequences of some mature cathelicidins are naturally resistant to serine proteases because these types of sequences are very poor substrates for proteolysis [10, 24]. In this study, we demonstrated that pro-prophenin 2 displays an acceptable stability after 1 h of interaction with* T. vaginalis* because protein degradation is not as evident as in our previous work with peptides derived from human cathelicidin LL-37 [28]. We did not test the stability of processed prophenin 2 after interaction with* T. vaginalis* because no antibody targeting the mature peptide was available.

## 5. Conclusions

We conclude that* T. vaginalis* integrity and growth were significantly diminished in the presence of prophenin 2. Although* T. vaginalis* secretes very active cysteine proteinases, pro-prophenin 2 partially overcomes this limitation, which is inherent to antimicrobial peptides, making prophenin 2 an interestingly candidate for alternative* T. vaginalis* treatment.
